# Response to letter regarding “ALVAC‐fIL2, a feline interleukin‐2 immunomodulator, as a treatment for sarcoids in horses: A pilot study”

**DOI:** 10.1111/jvim.16490

**Published:** 2022-07-20

**Authors:** Corey Saba

**Affiliations:** ^1^ Oncology Service, Small Animal Medicine & Surgery, Veterinary Teaching Hospital, College of Veterinary Medicine University of Georgia Athens Georgia USA

Dear Drs. Rishniw and White,

I appreciate your review and critique of our manuscript. The Veterinary Record article you referenced states: “Although widely accepted guidelines exist for how to conduct and report definitive clinical trials, there is no equivalent set of guidelines for pilot studies, the objectives of which are more variable. Within an increasing scholarly literature on pilot studies, consensus has emerged that pilot studies should focus on feasibility, process, and description, as opposed to group‐to‐group comparison of outcomes.” We believe that our study accomplished the goal of demonstrating feasibility and process. At the outset of the study, we recognized that it was a lot to ask of owners to trailer their horses to and from our hospital on a regular basis and worried that we would have difficulty with enrollment for this reason. However, with advertising, and once word got out that we were seeing positive responses, accruing horses was no problem at all. Admittedly, you are correct that we probably overstated our safety and efficacy results, as this was just a pilot study. However, on final review, Dr. DiBartola and I discussed this and opted to leave the conclusions as they were.

Sincerely,

Corey Saba

